# Structural valve deterioration is primarily caused by cyclic fatigue but not immune rejection

**DOI:** 10.3389/fimmu.2025.1652294

**Published:** 2025-09-03

**Authors:** Alexander E. Kostyunin, Tatiana V. Glushkova, Evgeny A. Ovcharenko, Pavel S. Onischenko, Kirill Yu. Klyshnikov, Tatiana N. Akentieva, Leo A. Bogdanov, Vladislav A. Koshelev, Alexander N. Stasev, Anton A. Khromov, Anton G. Kutikhin

**Affiliations:** ^1^ Division of Experimental Medicine, Research Institute for Complex Issues of Cardiovascular Diseases, Kemerovo, Russia; ^2^ Department of Cardiac and Vascular Surgery, Research Institute for Complex Issues of Cardiovascular Diseases, Kemerovo, Russia; ^3^ Radiology Department, Kuzbass Clinical Cardiological Center named after academician L.S. Barbarash, Kemerovo, Russia

**Keywords:** bioprosthetic heart valves, multivalvular replacement, cyclic loading, material fatigue, chronic immune rejection, immune cell infiltration, lipid retention, calcification

## Abstract

**Introduction:**

Currently, chronic immune rejection of bioprosthetic heart valves (BHVs) is considered among the key players in the development of structural valve degeneration (SVD). However, the relative contribution of leukocyte infiltration and cyclic mechanical loading into the SVD in bioprosthetic mitral valves (BMVs) and bioprosthetic tricuspid valves (BTVs, experiencing lower hemodynamic load due to the right heart’s pressure environment) remains unclear.

**Methods:**

Here we performed an investigation of BMVs and BTVs which have been pairwise-excised from 4 patients during the BHV replacement because of BMV failure. The amount of valvular calcification was measured by multislice computed tomography and quantified using Pydicom script. Immune cell infiltration and lipid deposition in sectioned leaflets were evaluated by hematoxylin and eosin and Oil Red O staining, respectively; the semi-quantitative analysis of whole slide images was conducted by QuPath and Fiji software. In addition, we conducted an ultrastructural examination of BHVs by backscattered scanning electron microscopy after epoxy resin embedding (EM-BSEM technique).

**Results and discussion:**

All BMVs had a significant extent of lipid deposition, hemorrhages, and tears, which eventually led to its mechanical incompetence. Strikingly, BMVs had less amount of immune cell infiltration as compared with BTVs. These results indicate that mechanical fatigue prevails over immune cell infiltration in driving the development of SVD.

## Introduction

Valvular heart disease (VHD) is among the leading causes of morbidity and mortality in elderly patients with cardiovascular disease (1), and its prevalence is predicted to rise because of the present trend to the population ageing (2). As current therapeutic approaches have not shown efficiency in VHD treatment, the replacement of the failing valve with mechanical heart valves (MHVs) or bioprosthetic heart valves (BHVs) remains the only appropriate option (3). While an average lifespan of MHVs varies from 20 to 30 years, their implantation is inevitably coupled with a lifelong intake of anticoagulants in order to avoid prosthetic valve thrombosis (4–6). In contrast, BHVs do not demand a significant anticoagulant treatment but their average longevity is limited to ≈ 15 years because of irreversible mechanical incompetence termed structural valve deterioration (SVD) (5, 7, 8). Repeated heart valve replacement is considered as a high-risk and unwanted event, although recent developments such as valve-in-valve procedure overcome the major complications to a certain extent (9).

Pathophysiology of SVD includes cyclic loading, enzymatic degradation, chronic immune rejection, and calcification (9, 10). Failing BHVs are generally notable for leukocyte infiltration, immunoglobulin deposition, and complement deposition (11–15). Macrophage infiltrations were primarily detected around the leaflet perforations, tears, and mineral deposits, suggesting antibody-mediated response and leukocyte infiltration as the primary causes of SVD (14, 15). However, co-localization of immune cells with the degraded extracellular matrix (ECM) and calcifications might indicate survivor’s mistake (i.e., dispensability) rather than a causative role of chronic immune rejection. The relative extent of leukocyte infiltration in bioprosthetic mitral valves (BMVs) and bioprosthetic tricuspid valves (BTVs), which experience a strikingly different hemodynamic load, also remains obscure. As such, the impact of chronic immune rejection into SVD pathogenesis needs a clarification.

Here we performed a detailed histological analysis of four BMVs and four BTVs excised pairwise from the same patients because of BMV failure. This study design permitted an objective comparison of the mechanisms behind the SVD in BMVs and BTVs, as both of these valves functioned in the identical immune and metabolic setup. Whilst all BMVs demonstrated a considerable lipid retention, multiple hemorrhages, and remarkable ECM degradation at high hemodynamic load, they displayed significantly less extent of leukocyte infiltration than BTVs which showed ECM integrity under the conditions of moderate hemodynamic load. This indicates an utmost importance of cyclic loading and a dispensable role of chronic immune rejection for the development of SVD.

## Materials and methods

### Study design

**Figure 1:**
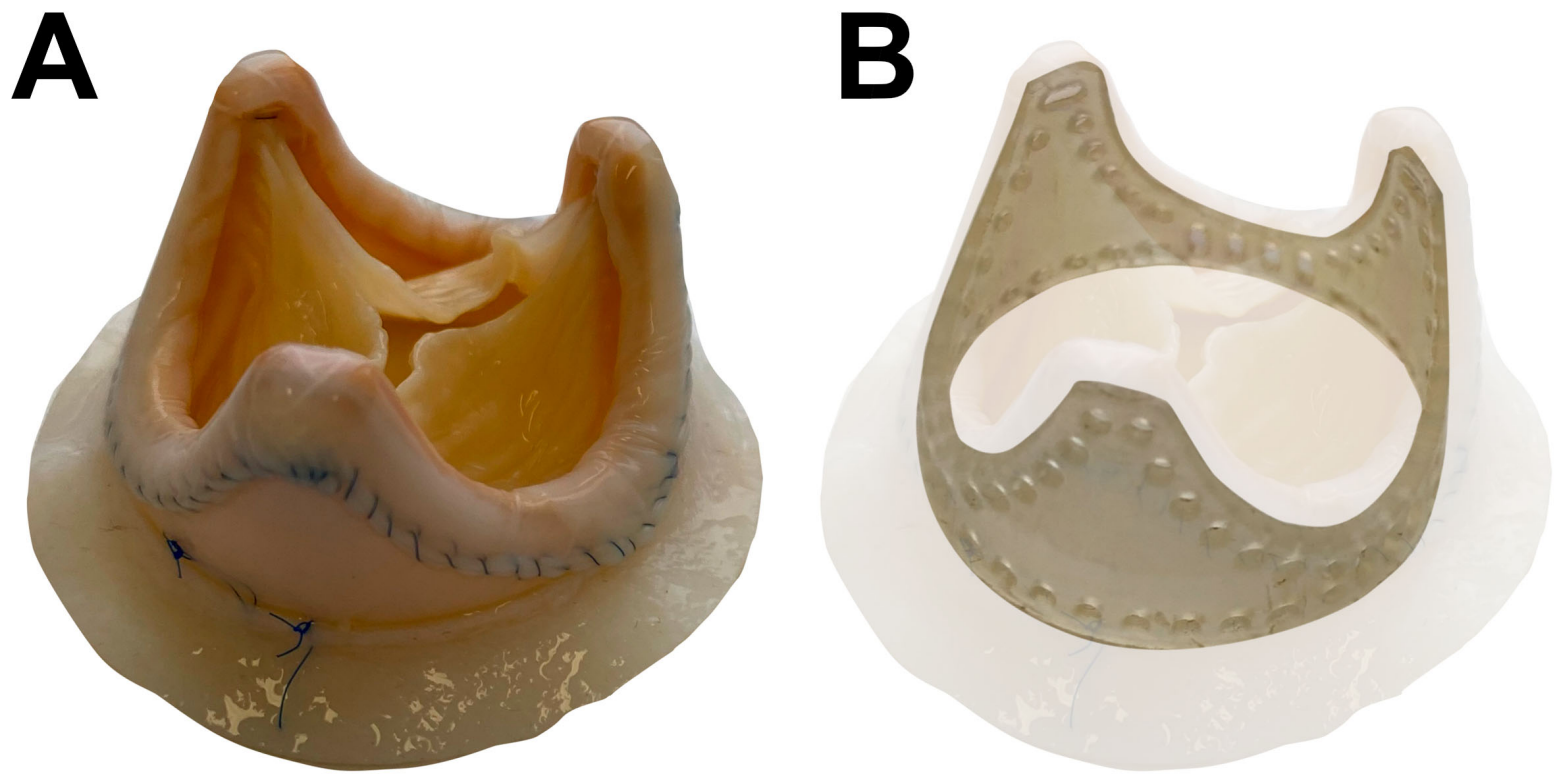
PeriCor bioprosthetic heart valve. **(А)** Overview. **(B)** Schematic visualization of supporting polypropylene frame.

Here we investigated 8 BHVs: BMVs (n = 4) and BTVs (n = 4) (KemCor or PeriCor devices, NeoCor, Kemerovo, Russia). KemCor and PeriCor are stented xenoaortic porcine BHVs designed for surgical implantation and manufactured from porcine aortic valves fixed with ethylene glycol diglycidyl ether instead of glutaraldehyde (**Figure 1**) (16, 17). Both KemCor and PeriCor models have three leaflets. The primary difference between KemCor and PeriCor is that supporting polypropylene frame of KemCor is covered with synthetic fabric, in contrast to PeriCor which employs calf pericardium for better biocompatibility and higher resistance to infective endocarditis. Manufacturing of KemCor and PeriCor has been terminated in 2001 and 2009, respectively. However, these BHV models can be used for studying the pathophysiology of BHV failure to improve the design of next-generation BHVs.

During the primary heart valve replacement because of rheumatic heart disease, each of the 4 patients received identical BHVs (either KemCor or PeriCor) for the mitral and tricuspid positions, which were then pairwise excised during the repeated heart valve replacement because of BMV failure. The diagnosis [stage III SVD for BMVs and stage I for BTVs (8)] has been made after the echocardiographic examination. All BTVs retained their function by the time of the intervention, yet have also been replaced to prevent further development of SVD. Hence, BMVs and BTVs were implanted and excised concurrently in each patient, and the duration of functioning was identical for both valves. The same surgical incision was used to obtain an access to BMVs and BTVs for performing a multivalvular replacement. Specimen collection was approved by the Local Ethical Committee of the Research Institute for Complex Issues of Cardiovascular Diseases (ethical approval code 16/2024, approved on 16 August 2024), and a written informed consent was provided by all study participants after receiving a full explanation of the study. The investigation was carried out in accordance with the Good Clinical Practice and a latest revision of the Declaration of Helsinki (2013). Clinicopathological features of patients and BHVs are provided in **Tables 1**, **2**, respectively.

**Table 1:** Clinicopathological features of patients who underwent repeated heart valve replacement.

Patient	Age at primary heart valve replacement, years	Gender, male/female	Arterial hypertension	Dyslipidemia	Overweight or obesity	Type 2 diabetes mellitus	Chronic kidney disease	New York Heart Association functional class of chromic heart failure
Patient #1	57	M	–	–	+	–	–	IV
Patient #2	59	M	+	–	+	–	–	III
Patient #3	42	F	–	–	–	–	–	II
Patient #4	66	F	+	–	–	–	–	IV

**Table 2:** Clinicopathological features of excised BHVs.

Patient	Implantation position	Lifespan, months	BHV type	Diagnosis
Patient #1	Mitral	147	KemCor	Mitral regurgitation grade IV
	Tricuspid	147	KemCor	Normal function
Patient #2	Mitral	108	PeriCor	Mitral regurgitation grade IV
	Tricuspid	108	PeriCor	Stenosis grade I
Patient #3	Mitral	215	KemCor	Mitral regurgitation grade III
	Tricuspid	215	KemCor	Stenosis grade I
Patient #4	Mitral	120	PeriCor	Mitral regurgitation grade III
	Tricuspid	120	PeriCor	Paravalvular leak grade II

### Quantification of calcification

Calcification of BHVs was evaluated by multislice computed tomography (MSCT, LightSpeed VCT 64, General Electric, Boston, MA, USA) using the following parameters: tube voltage 120 kV; tube current 160 mA; gantry rotation time 0.9 seconds; scanning time 6.8 seconds; table feed speed 39.37 mm per rotation. 3D reconstruction was conducted utilizing a slide thickness of 0.625 mm. Images were formed using a standard kernel.

Digital Imaging and Communications in Medicine (DICOM) files were analyzed employing a Pydicom package (Python 3.9, Python Software Foundation, Wilmington, DE, USA). All MSCT layers were united into a 3D array where XY plane contained the parameters of brightness for each pixel along the Z axis. The volume of each BHV and its calcium deposits were defined using a pixel brightness threshold (400 units for pericardium and 1700 units for calcium deposits). To quantify calcification volume, we reconstructed a 3D binary array using a calcification mask for each BHV. In such array, pixel brightness was quantified to 1 if calcification mask pixel was not 0. Next, the number of such pixels was multiplied by the volume of the image voxel to obtain the total volume of calcium deposits within the BHVs. The parameters of the image voxel were calculated utilizing the metadata of DICOM files. The verification of such values was performed in Materialize Mimics software (Materialize NV, Leuven, Belgium) with < 1% error. 2D images of calcium deposits were represented as an average of brightness amongst all layers of the 3D image.

### Histological analysis

For the histological analysis, we dissected the leaflet fragments in the central and commissural zones (from the base to the free margin) and in the fragments with pathological thickening, tears, or calcium deposits). Selected fragments were snap-frozen (Epredia Neg-50 Frozen Section Medium, 6502, Thermo Fisher Scientific, Waltham, MA, USA) and sectioned (6 µm thickness) using Microm HM525 cryostat (Thermo Fisher Scientific, Waltham, MA, USA). To conduct a general histological examination, sections were then stained with Gill’s hematoxylin (HK-G0-BL01, ErgoProduction, Saint Petersburg, Russia) for 10 minutes, rinsed in tap water for 5 minutes, immersed in distilled water, and incubated in alcoholic eosin Y solution (HK-ES-BL01, ErgoProduction, Saint Petersburg, Russia) for 1 minute. After the brief washing in distilled water and three changes in 95% ethanol (Kemerovo Pharmaceutical Plant, Kemerovo, Russia), sections were cleared with xylene (HP-XR-1L01, ErgoProduction, Saint Petersburg, Russia) for 3 minutes. Coverslips were mounted with Vitrogel (12-005, ErgoProduction, Saint Petersburg, Russia). Collagen content and lipid deposition were assessed by Russell-Movat’s pentachrome staining (ab245884, Abcam, Cambridge, UK) and Oil Red O staining (ab150678, Abcam, Cambridge, UK), respectively, according to the manufacturer’s protocols. For the Oil Red O staining, coverslips were mounted with 50% glycerol (8.06.00284, ChemExpress, Ufa, Russia). Whole slide images were acquired using (Vision Slide Assist, West Medica, Perm, Russia). Image post-processing and semi-quantitative image analysis (i.e., cell count and area measurements) were carried out in QuPath software v.0.5.1 (18). For the quantification of immune cell infiltration and lipid deposition, we selected 10 representative sections from each BHV (3 or 4 sections per each leaflet). Immune cell density was counted at both inflow and outflow sides (100 µm thickness) as well as in the depth of the leaflet. Lipid deposition was measured as a proportion of Oil Red O-positive area within the leaflet to the total leaflet area using Fiji software (National Institutes of Health, Bethesda, MD, USA) (19). As these BHVs were stored in 10% neutral phosphate-buffered formalin for ≥ 8 years, immunohistochemistry and Western blotting have not been applied in this study.

### Ultrastructural analysis

Ultrastructural analysis of excised BHVs was carried out by a backscattered scanning electron microscopy after epoxy resin embedding (EM-BSEM technique) (20). Leaflet fragments were fixed in 2 changes (12 hours per each) of 10% neutral phosphate-buffered formalin (B06-003, ErgoProduction, Saint Petersburg, Russia) at 4°C. Then, leaflets were washed in three changes (10 minutes per each) of phosphate-buffered saline (PBS, 0.1 mol/L, pH 7.4), postfixed in 1% phosphate-buffered osmium tetroxide (OsO_4_, 19110, Electron Microscopy Sciences, Hatfield, PA, USA) for 16 hours and stained with 2% aqueous osmium tetroxide for 40 hours. Next, leaflets were washed in four changes (15 minutes per each) of PBS, dehydrated in ascending ethanol series (50%, 60%, 70%, 80%, and 95%, two changes per each concentration, 15 minutes per change), stained in 2% alcoholic uranyl acetate (22400-2, Electron Microscopy Sciences, Hatfield, PA, USA) for 16 hours, dehydrated in two changes (15 minutes per each) of 95% ethanol, isopropanol (06-002, ErgoProduction, Saint Petersburg, Russia) for 2 hours, and acetone (6-09-20-03-83, EKOS-1, Moscow, Russia) for 2 hours. After the dehydration, leaflets were impregnated with a blend of acetone and epoxy resin (1:1) for 16 hours and pure epoxy resin (Araldite 502, 13900, Electron Microscopy Sciences, Hatfield, PA, USA) for 24 hours, and embedded into the fresh epoxy resin at 60°C for another 24 hours. Following the epoxy resin polymerization, leaflets were grinded and polished (TegraPol-11, Struers, Copenhagen, Denmark) and counterstained with Reynolds’s lead citrate (17810, Electron Microscopy Sciences, Hatfield, PA, USA). After the washing in three changes (5 minutes per each) of distilled water, leaflets were sputter coated with carbon (10 nm thickness, Leica EM ACE200, Leica Microsystems, Wetzlar, Germany), and visualized by backscattered scanning electron microscopy (BSECOMP mode, S-3400N, Hitachi, Tokyo, Japan) at high vacuum and 10 or 15 kV accelerating voltage.

### Statistical analysis

Statistical analysis was performed using GraphPad Prism 8 (GraphPad Software, San Diego, CA, USA). For descriptive statistics, data were represented by the proportions, the median, and 25th and 75th percentiles. Groups were compared by the Mann-Whitney U-test. *P* values ≤ 0.05 were regarded as statistically significant.

## Results

### Pairwise comparison reveals material fatigue and calcification in BMVs but not BTVs

**Figure 2:**
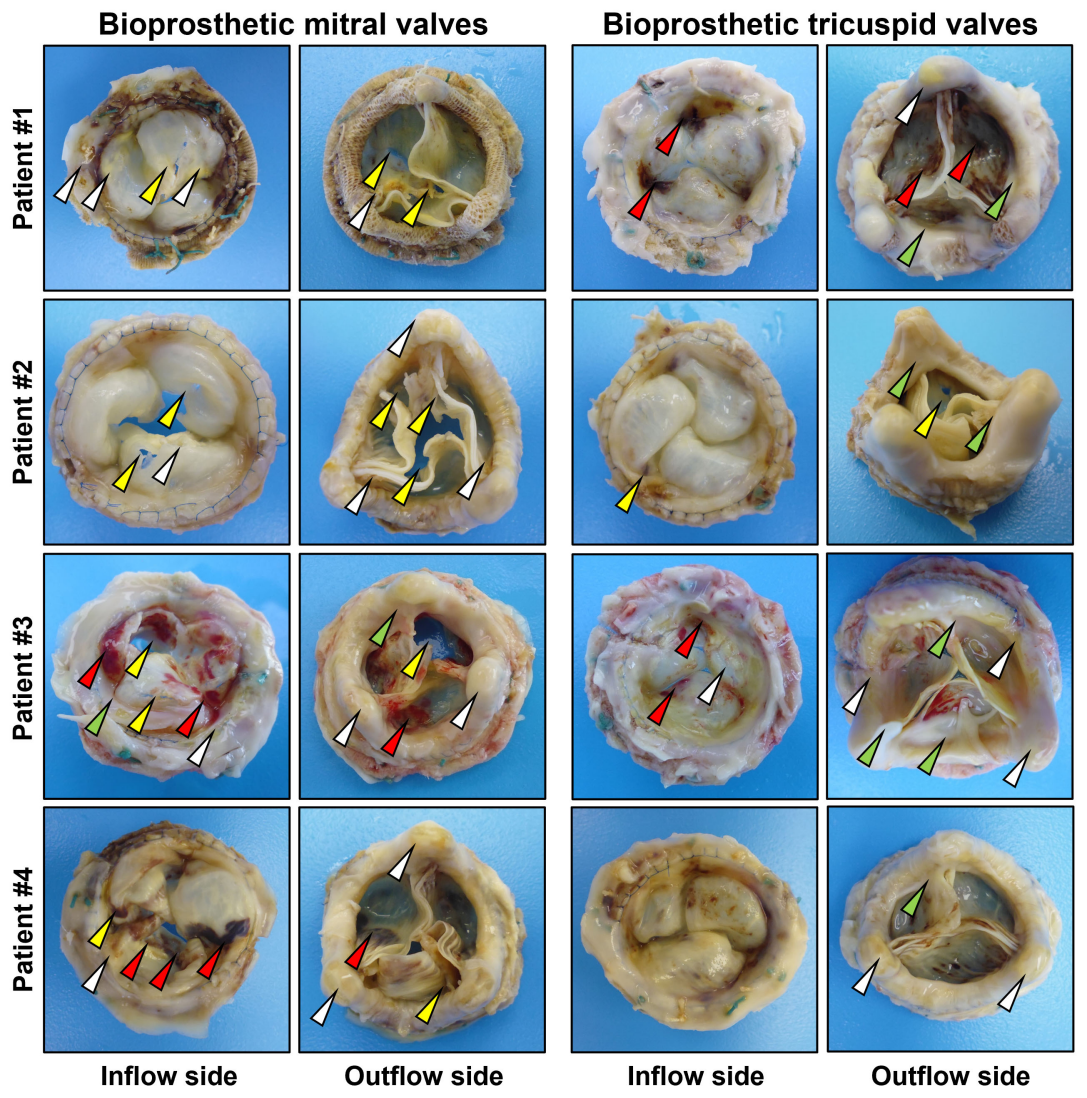
Excised bioprosthetic mitral valves (BMVs, left) and bioprosthetic tricuspid valves (BTVs, right). White, yellow, red and green arrows indicate calcium deposits, perforations/tears, intravalvular hemorrhages, and pannus.

**Figure 3:**
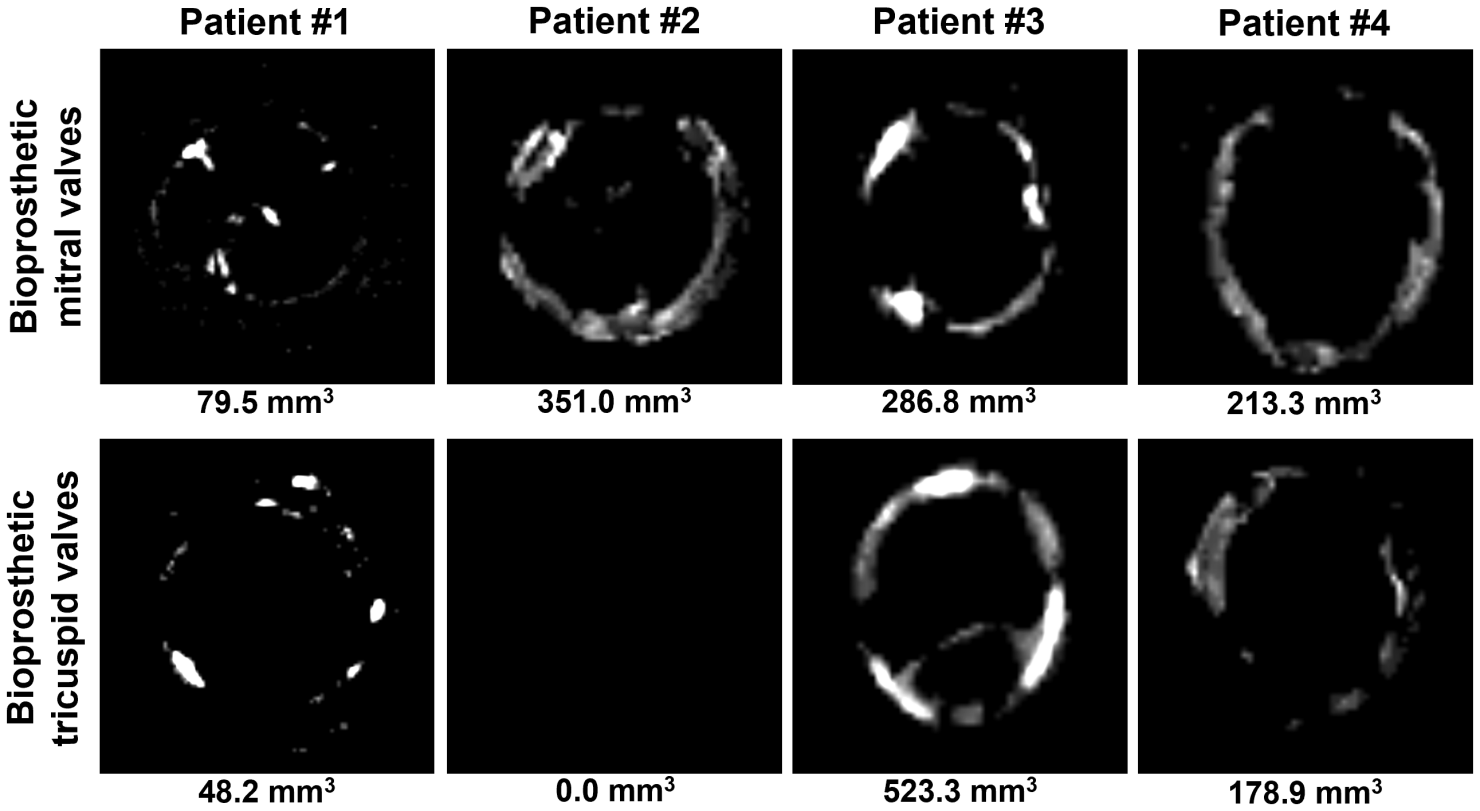
Visualization of calcium deposits in bioprosthetic mitral valves (BMVs, top) and bioprosthetic tricuspid valves (BTVs, bottom) by an averaged 2D projection of multislice computed tomography images. Calcification volume is indicated below each image.

All BMVs had perforations and tears localized primarily in the dome of the leaflets and around the commissures, and two BMVs (patients #3 and #4) had large intravalvular hemorrhages (**Figure 2**). In contrast, only one BTV (patient #2) had minor perforation, whilst two BTVs (patients #1 and #3) had small intravalvular hemorrhages (**Figure 2**). All BTVs and one BMV (patient #3) had a moderate pannus (i.e., a pathological outgrowth of the fibrotic tissue) at the base and commissural areas at the outflow side (**Figure 2**). In two BTVs (patients #2 and #3), pannus restricted the valve opening, accounting for grade I stenosis revealed at echocardiography. All BMVs and BTVs suffered from minor calcification in the xenopericardial coating of the polypropylene frame that did not impact BHV functioning. In 3 out of 4 pairs (patient #1, patient #2, and patient #4), BMVs had higher amounts of calcium deposits than BTVs (**Figure 3**). Hence, failing BMVs had typical signs of material fatigue because of higher hemodynamic load, whereas BTVs retained integrity.

### BMVs are more prone to lipid infiltration than BTVs, which exhibit more pronounced cellular infiltration

**Figure 4:**
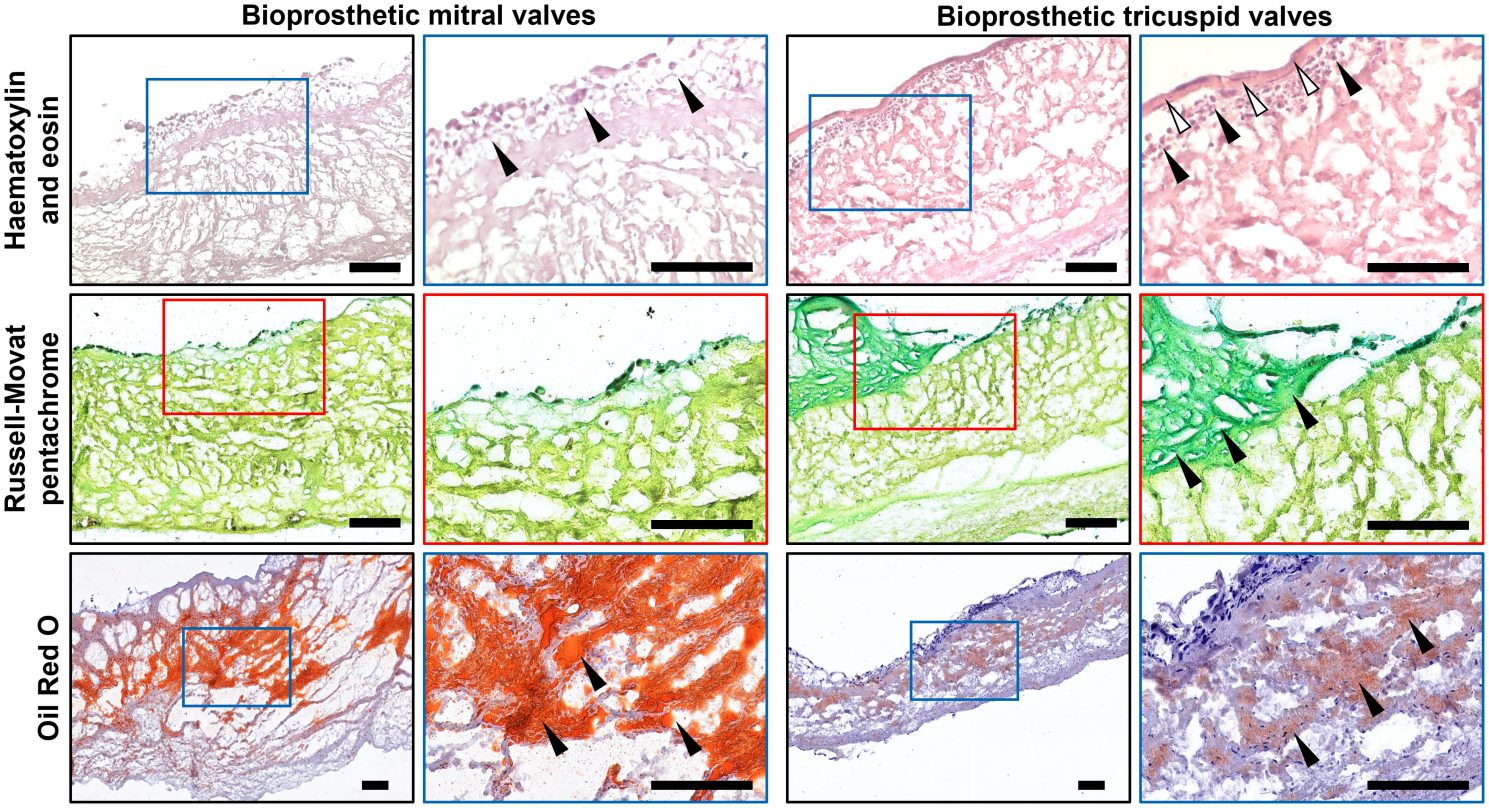
Histological analysis of bioprosthetic mitral valves (BMVs, left) and bioprosthetic tricuspid valves (BTVs, right). Hematoxylin and eosin staining (top) indicates the host cells (indicated by black arrows) at and beneath the inflow or outflow side (white arrows indicate the pannus). Russell-Movat’s pentachrome staining (middle) shows collagen (yellow color) but not glycosaminoglycans (green color) in the bioprosthetic ECM, also showing abundant glycosaminoglycans (green color) within the pannus (indicated by black arrows), in particular in BTVs. Oil Red O staining (bottom) detected lipid droplets, fatty spots, and fatty streaks (indicated by black arrows) mostly in the spongiosa layer. Note significantly higher lipid retention in BMVs in comparison with BTVs. Scale bars: 100 μm.

Hematoxylin and eosin staining found sporadic cellular infiltrations in BMVs and BTVs (**Figure 4**). Most of the cells were located at and beneath the inflow or outflow side at 50-100 µm depth, or within the areas of degraded ECM. In BMVs, cells were also detected along the edges of perforations and tears; in BTVs, dense cell layer was found between the xenopericardium and pannus (**Figure 4**). Russell-Movat’s pentachrome staining identified abundant amounts of glycosaminoglycans within the pannus that confirmed its synthesis by the host cells, whereas bioprosthetic ECM was devoid of ground substance (**Figure 4**). None of the BHVs contained fibrin at the inflow or outflow side. Oil Red O staining detected numerous lipid droplets in the spongiosa layer and beneath the free margin of BMVs and BTVs (**Figure 4**). Notably, BMVs had multiple fatty spots and fatty streaks which were absent in BTVs.

**Figure 5:**
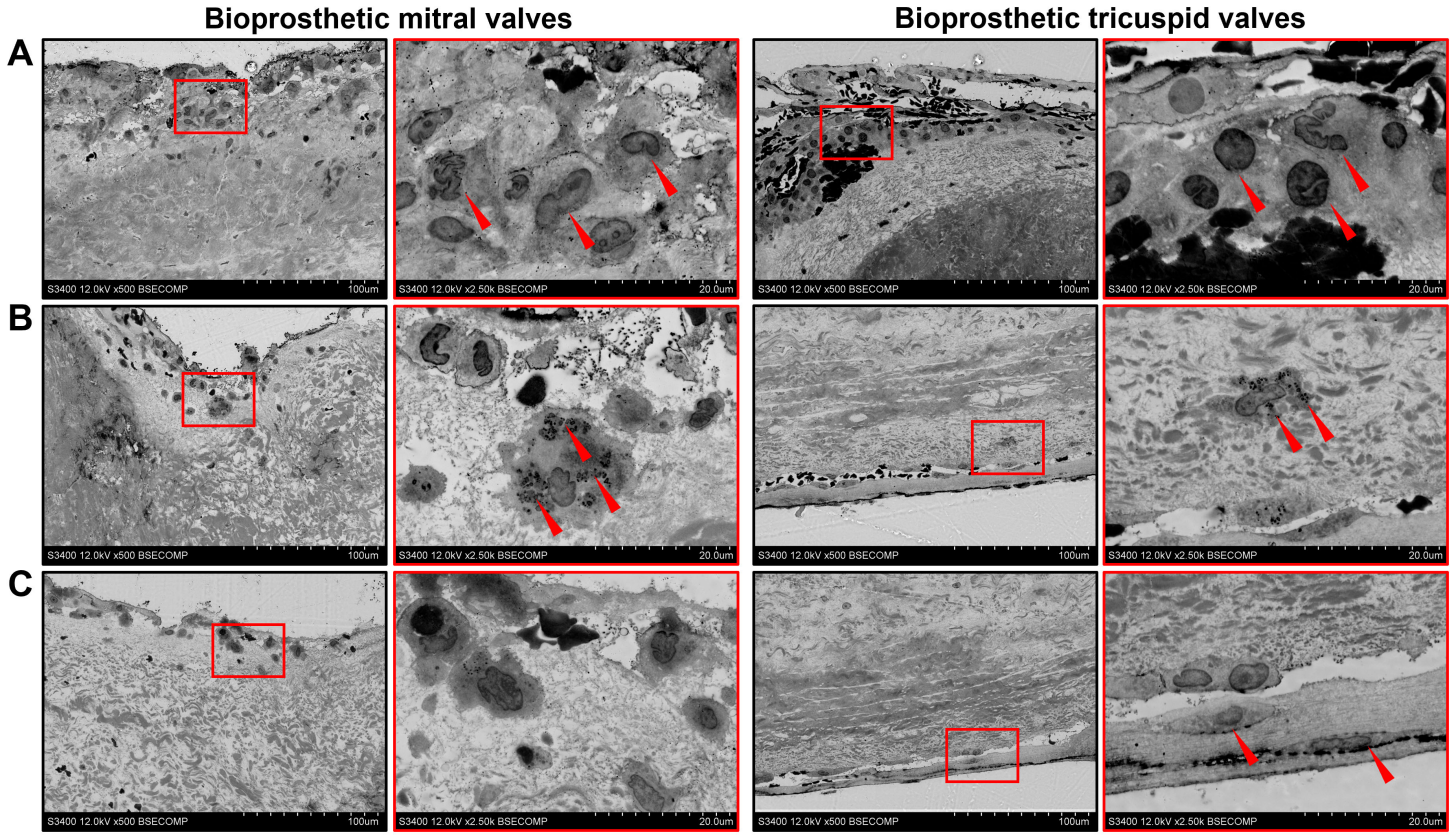
Ultrastructural analysis of bioprosthetic mitral valves (BMVs, left) and bioprosthetic tricuspid valves (BTVs, right). **(A)** Macrophages (indicated by red arrows) constitute the majority of host cells beneath the inflow or outflow side. **(B)** A significant proportion of macrophages had notable matrix-degrading activity as evident by electron-dense granules on their cytoplasm (indicated by red arrows). **(C)** Myofibroblasts (indicated by red arrows) within the pannus in BTVs.

Having performed an electron microscopy analysis of BHV leaflets, we recognized the most of the infiltrating host cells as macrophages, foam cells, and multinucleated giant cells (**Figures 5A, B**). Macrophages and multinucleated giant cells frequently contained electron-dense cytoplasmic inclusions suggestive of ECM degradation (**Figure 5C**). In addition to macrophages, BTVs also contained myofibroblasts in the pannus (**Figure 5C**).

**Figure 6:**
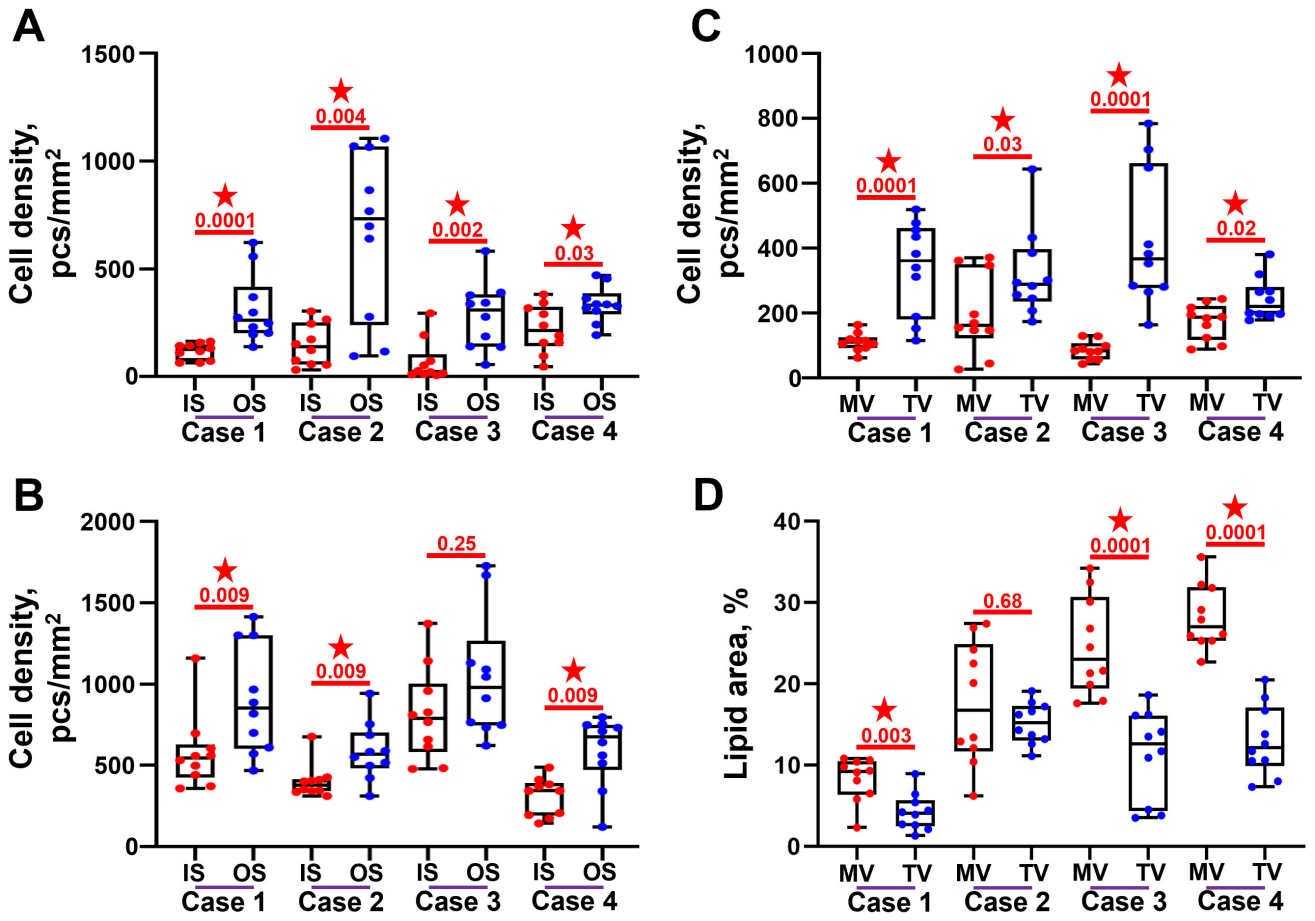
Semi-quantitative analysis of cell infiltration and lipid deposition in bioprosthetic mitral valves (MV) and bioprosthetic tricuspid valves (TV). **(A)** Cell density at and beneath (up to 100 µm depth) the inflow side (IS) and outflow side (OS) in the BMVs. **(B)** Cell density at and beneath (up to 100 µm depth) the inflow side (IS) and outflow side (OS) in the BTVs. **(C)** Total cell density within the leaflets of BMVs and BTVs. **(D)** Proportion of Oil Red O-positive area within the leaflets of BMVs and BTVs. Statistically significant differences are marked by a red star.

Cell density at the outflow side was higher than at the inflow side in both BMVs (**Figure 6A**) and BTVs (**Figure 6B**). In all BHV pairs, total cell density was higher in BTVs than in BMVs (**Figure 6C**). In 3 out of 4 pairs, the area of lipid deposition was higher in BMVs as compared with BTVs, in keeping with the previous findings on increased ECM degradation and disintegration in BMVs (**Figure 6D**).

## Discussion

In each of four cases included in this study, repeated heart valve replacement was carried out because of BMV failure, whereas BTVs were free from dysfunction or had mild dysfunction. In line with the echocardiographic data, gross examination and histological analysis revealed that, as opposed to the BTVs, BMVs had critical defects such as intravalvular hemorrhages, perforations/tears, and calcification. Our results are in concord with Cohen and colleagues who performed a concurrent implantation of Hancock bioprostheses into the distinct atrioventricular positions and found accelerated SVD in the BMVs after the excision of both BMVs and BTVs (21). Similar results were obtained by Ohata and colleagues who conducted a 13-year follow-up and documented that freedom from SVD was 78 ± 22% and 100%, and freedom from the repeated replacement was 70 ± 30% and 90% ± 10% for BMVs and BTVs, respectively (22). We and others (23) suggest that the differences in the rates of SVD between BMVs and BTVs in the same patients can be explained solely by distinct hemodynamic load, as other factors such as immune response and metabolic activity are shared by both atrioventricular positions.

Systolic blood pressure (from 100 to 120 mmHg) and transvalvular jet velocity (from 0.6 to 1.3 m/s) in the left ventricle strikingly exceed those in the right ventricle (from 20 to 25 mmHg and from 0.3 to 0.7 m/s, respectively), indicating a higher mechanical stress exerted on BMVs (24–27). The role of cyclic fatigue in accelerating SVD has been indicated earlier during the examination of HeartMate VE left ventricular assist system which employs two identical porcine valves (inflow and outflow) (23). Inflow valves experience higher pressure in comparison with outflow valves, more frequently suffer from calcification and have higher amount of calcium deposits (23). As immune and metabolic setups are common for both atrioventricular positions (i.e., mitral and tricuspid valves), differences between BMVs and BTVs can be explained solely by distinct hemodynamic load. Having taken into account both the abovementioned arguments (i.e., reference ranges from the literature, HeartMate VE case (23), and our examination of identical BMVs and BTVs within the same patient), here we provide collective evidence for the higher physiological pressure in mitral than in tricuspid valve position. Both HeartMate VE study (23) and our investigation can be considered as two clinical scenarios (i.e., implantable left ventricular assist device with inflow and outflow BHVs and distinct atrioventricular BHVs) confirming the theoretical background.

As multivalvular BHV replacements are relatively rare even in specialized centers, we were unable to collect other BHVs excised pairwise from aortic and mitral, or aortic and tricuspid positions, and this is the study limitation. Comparison of BMVs (or BTVs) with bioprosthetic aortic valves (BAVs) obtained from different patients do not exclude immune and metabolic background and therefore has not been conducted in this study. Another point is that atrioventricular and aortic BHVs have different size and design, hence being inherently prone to distinct deformation profiles. Evolutionarily, native MV has been adapted to the indicated hydrodynamic conditions (28, 29). Anterior MV leaflet is thicker than the corresponding TV cusp (≈ 0.79 vs. ≈ 0.52 mm, respectively) and has higher collagen content (≈ 77 vs. ≈ 68%, respectively) (30, 31). Along similar lines, porcine TVs have lower elastin and glycosaminoglycan content as compared with MVs (31, 32). As opposed to native HVs, BMVs do not have any constructive features aimed at withstanding a challenging hemodynamic load. Mechanical stress induces material fatigue and irreversible degradation of the prosthetic ECM, ultimately leading to the ruptures and calcification (33–35) that have been observed in the BMVs.

Because of the high systolic blood pressure in the left heart, BMVs suffered from numerous intraleaflet hemorrhages and lipid deposition. Presumably, BMVs should also contain increased amounts of serum proteins (i.e., albumin, osteopontin, fibrinogen, and matrix metalloproteinase 9 (MMP-9) (36–39). In combination with material fatigue, such deposition of ECM-degrading and calcium-binding molecules could explain accelerated degeneration of BMVs. Unfortunately, BMVs and BTVs interrogated in this study have been stored in formalin for ≥ 8 years, restricting the opportunities for immunodetection.

Strikingly, BMVs were less affected by immune cell infiltration than BTVs, probably due to the less turbulent flow and lower shear stress in the right heart which foster cell adhesion (40). In both BMVs and BTVs, host cells prevailed in the outflow side in comparison with the inflow side, supporting this suggestion. High shear stress in the left heart and in the inflow side of both BMVs and BTVs may preclude cell adhesion to the cusps or shed the majority of adhered cells.

Besides inflow and outflow sides, host cells were focused in the areas of degraded ECM, in particular around the perforations and tears within the BMVs. This corresponds to the previous findings which reported the co-localization of immune cells with the fragmented ECM (14, 15, 37, 38). Previously, it has been shown that macrophage infiltration promotes SVD via release of proteolytic enzymes (such as matrix metalloproteinases and cathepsins), reactive oxygen species, and pro-calcific extracellular vesicles (14, 15, 37, 38, 41). Macrophages might digest and engulf even chemically cross-linked ECM fibers (42). Nevertheless, our results do not support the hypothesis on the leading role of chronic immune rejection in SVD pathogenesis (9, 10, 14, 15). Despite an immune cell attack, BTVs were relatively resistant to SVD and retained their function. Earlier, we have also shown that xenoaortic porcine BHVs, which are prone to leukocyte infiltration, had a higher lifespan that xenopericardial bovine BHVs with a mild-to-moderate amounts of immune cells (37). Intriguingly, both indicated BHV types had similar levels of blood-derived MMP-9 (37). Taken together, these observations suggest that cyclic loading and precipitation of circulating proteases and calcium-binding proteins are more significant for SVD that macrophage invasion. Immune response, however, might take a leading role in case of hypersensitivity to the xenogeneic antigens (43).

Albeit resistant to SVD, BTVs exhibited a pathological outgrowth of host-derived connective tissue at their surfaces (i.e., pannus), that was not the case for the BMVs. Pannus typically contains neutrophils, macrophages, multinucleated giant cells, myofibroblasts, and mast cells (44) and grows upon the contact of host tissues with the metal surfaces or bioprosthetic tissues (45–50), representing a case of aseptic inflammation- and transforming growth factor β (TGF-β)-driven foreign body reaction (9, 12, 44, 51). Macrophage-derived TGF-β controls proliferation and differentiation of ECM-producing mesenchymal cells such as myofibroblasts, sustaining physiological regeneration or inducing pathological fibrosis (52, 53). Patients with pannus generally have elevated levels of plasma TGF-β as compared with those without (54). A remarkable immune cell infiltration observed in BTVs partially explains a pannus formation; further, moderate outgrowth of host tissue reduces thrombogenicity of BHVs and affirms their attachment (54). However, excessive formation of pannus might induce prosthetic valve stenosis and cause its dysfunction regardless of SVD (55–57).

## Conclusions

Pairwise comparison of BMVs and BTVs revealed that the BMVs suffered from intravalvular hemorrhages, contained multiple lipid spots/streaks, and acquired structural defects (i.e., perforations and tears) but had fewer immune cells in comparison with the BTVs. The indicated differences between the BMVs and BTVs can be explained by higher systolic blood pressure, transvalvular jet velocity, and significant shear stress in the mitral position, which collectively enhance material fatigue, promote ECM degradation, and interfere with cell adhesion. In spite of higher leukocyte infiltration, BTVs retained their function and did not have histological features of SVD. We concluded that cyclic fatigue is a key driver of SVD, whilst chronic immune rejection is of lesser concern.

## Data Availability

The original contributions presented in the study are included in the article/supplementary material. Further inquiries can be directed to the corresponding author.
